# Undernutrition and associated factors among adult prisoners in Fiche town, central Ethiopia: a facility-based cross-sectional study

**DOI:** 10.3389/fnut.2023.1144654

**Published:** 2023-07-04

**Authors:** Mengistu Wondimu, Ayichew Siyoum, Indeshaw Ketema, Abel Tibebu Goshu, Sisay Habte, Ame Mehadi, Behailu Hawulte Ayele

**Affiliations:** ^1^Ejere Health Center, Hidebu Abote Woreda, North Shoa Zone, Fiche, Ethiopia; ^2^School of Medical Laboratory Sciences, College of Health and Medical Sciences, Haramaya University, Harar, Ethiopia; ^3^School of Nursing and Midwifery, College of Health and Medical Sciences, Haramaya University, Harar, Ethiopia; ^4^School of Public Health, College of Health and Medical Sciences, Haramaya University, Harar, Ethiopia

**Keywords:** undernutrition, associated factors, prisoners, Fiche, Ethiopia

## Abstract

**Background:**

Undernutrition is a major public health problem worldwide, particularly in developing countries like Ethiopia. However, nutritional problems are frequently overlooked in low-income countries, especially among vulnerable populations such as imprisoned people. The scientific data on the rate of undernutrition among imprisoned people in Ethiopia is limited. Hence, this study aimed to assess the magnitude and associated factors of undernutrition among adult prisoners in Fiche town, central Ethiopia.

**Methods:**

A facility-based cross-sectional study was conducted from August 15 to September 15, 2020. A systematic random sampling technique was used to select participants. All prisoners whose age was 18 years and above who have been in prison for at least 6 months were included. Data were collected using interviewer-administered pretested semi-structured questionnaires and standard anthropometric measurements. A cut-off point of body mass index <18.5 kg/m^2^ was used to measure undernutrition. Data were coded, entered into Epi-data version 3.1, and analyzed using Statistical Package for Social Sciences version 20.0. A binary logistic regression analysis was conducted to identify factors associated with undernutrition. The adjusted odds ratio (AOR) with a 95% confidence interval (CI) was calculated to measure the strength of the association and a *p*-value of less than 0.05 was considered statistically significant.

**Results:**

The overall magnitude of undernutrition among adult prisoners was 20% (95% CI: 16.5–23.6). Duration of imprisonment, incarcerated for 25 to 59 months (AOR = 3.07; 95% CI: 1.33, 7.04) and for greater than 59 months (AOR = 4.56; 95% CI: 2.0, 10.45), mild and moderate depression (AOR = 1.9; 95% CI: 1.05, 3.45), and moderately severe and severe depression (AOR = 2.78; 95% CI: 1.17, 6.60) were significantly associated with increased odds of undernutrition. However, being female (AOR = 0.51; 95% CI: 0.26, 0.98), having financial support (AOR = 0.36; 95% CI: 0.15, 0.87), engaging in income-generating work within the prison (AOR = 0.27; 95% CI: 0.15, 0.47), having medium dietary diversity (AOR = 0.35; 95% CI: 0.15, 0.80), and having good dietary diversity (AOR = 0.23; 95% CI: 0.08, 0.61) significantly decreased the odds of undernutrition.

**Conclusion:**

The magnitude of undernutrition among adult prisoners was high, with one in five prisoners in Fiche town prison having undernutrition. Sex, financial support, duration of imprisonment, income-generating work in the prison, dietary diversity, and depression were predictors of undernutrition. Hence, access to healthy food and diversified diets should be ensured for prisoners, and implementing early screening and treatment of depression, as well as encouraging prisoners to engage in income-generating work within the prison is recommended to reduce the burden of undernutrition.

## Introduction

Malnutrition refers to either inadequate intake of nutrients or intake of nutrients over body requirements (1, 2). The rate of malnutrition is high in developing countries, ranging from 6% to 48% among older adults in sub-Saharan Africa (2). In Ethiopia, malnutrition is a common health problem affecting 21.9% of the population in 2014 (3). Factors such as inadequate food consumption, increased nutrient loss, inadequate nutrient absorption, loss of appetite, inability to chew and swallow, and rising prescription drug use are identified as being associated with malnutrition (4–6). The health consequences of malnutrition are significant, ranging from delayed recovery to increased mortality (7, 8). Insulin resistance, poor immune function, reduced muscle strength, difficulty keeping warm, dyslipidemia and diminished capacity for hard labor are possible physiologic consequences of malnutrition (9–11).

Globally, nearly 11 million people are confined in prisons, with the largest number held in Africa (12). Marginalized and poor people make up a large percentage of the world’s prisoners (13). There are approximately 100,000 to 120,000 inmates in Ethiopia (14). Nowadays, due to the increasing number of imprisoned people, the provision of adequate food remains an ongoing problem, putting prisoners at risk of malnutrition (15). Prisoners are subjected to a variety of harmful and deterrent health conditions, including nutritional-related problems (16, 17).

Access to adequate nutrition is a basic human right and prisoners should be provided with healthy food choices and diversified diets to optimize health (18, 19). Prisoners are exposed to a number of unfavorable health and health-deterring factors including higher risks of mortality and injuries (15–17, 20, 21). The literature indicates that prisoners face different nutritional problems ranging from several micronutrient deficiencies and re-emerging related diseases to delayed recovery, mental illness, sexual health problems, infectious diseases, and increased risk of mortality (22–24).

Prisoners are particularly vulnerable to suffering from undernutrition compared to the general population (7, 25). Several factors have been identified for undernutrition among prisoners, including lack of adequate food, poor dietary diversity, infections, prolonged duration of imprisonment, absence of financial support and family visits, and overcrowding living conditions (26–28). In addition, behavioral factors such as smoking, khat chewing and alcohol use are identified risk factors of undernutrition (29).

In Africa, there is limited evidence on the various health problems of prisoners despite the relevance of such evidence to the health of the prisoners and the community (29). Prisoners incarcerated in developing countries are especially vulnerable to dietary deficiencies, with the highest rate in the prisons of low-income countries (8). For instance, 38.4% of the female detainees in Antanmora prison, Madagascar were undernourished (28). The evidence from two studies in Ethiopia indicated that about 18.6% and 23.2% of adult prisoners were undernourished (30, 31).

In low-income countries, nutrition-related issues are often neglected; particularly among vulnerable groups such as imprisoned people (32). The foods served in many prisons are not sufficient in terms of quantity and quality (33). In many developing countries, including Ethiopia, the incarcerated people primarily obtain their food from the prison, which is insufficient to meet their nutritional needs (7). In Ethiopia, the prison health system is not well integrated with the national health system, and the health problems of prisoners are mostly marginalized by researchers (29). Hence, the scientific data on the rate of undernutrition among prisoners in Ethiopia is limited. Therefore, this study aimed to determine the magnitude and predictors of undernutrition among adult prisoners in Fiche town, central Ethiopia.

## Methods and materials

### Study setting and period

The study was conducted in Fiche town prison, North Shewa, central Ethiopia from August 15 to September 15, 2020. Fiche town is located 152 km from Addis Ababa, the capital city of Ethiopia, to the north. The town is the administrative center for the North Shewa Zone of Oromia Regional State. Fiche Town Prison serves as a central destination for inmates coming from surrounding smaller prisons or police stations. There were 2,100 prisoners in Fiche town prison during the study period, of whom 1,200 (749 males and 451 females) were adults 18 years of age and older.

### Study design and population

A facility-based cross-sectional study was conducted to determine the magnitude of undernutrition and associated factors among adult prisoners in Fiche town prison, central Ethiopia. All randomly selected prisoners whose age was 18 years and above who have been in prison for at least 6 months and were available during the data collection period were included in the study. Whereas, pregnant and lactating mothers as well as prisoners with physical deformities who were unable to assume an erect position during anthropometric measurements were excluded.

### Sample size determination

The sample size required for the study was determined using a single population proportion formula with the assumptions of a 25.2% prevalence of undernutrition among adult prisoners taken from a previous study conducted in Northern Ethiopia (29), a 95% CI, and a 4% margin of error. Hence, the final sample size required for this study after adding a 10% for non-response rate was **497**.

### Sampling techniques and procedures

A stratified random sampling technique was employed to form the strata by the sex of eligible prisoners. A sampling frame was created using lists of eligible prisoners obtained from the prison administration office. Then, after stratifying the sampling frame by the sex of prisoners, the estimated sample size was proportionally allocated to the size of each stratum. The study sample was selected using prisoners’ identification (ID) numbers. The first participant was selected by lottery method from each stratum. Finally, the study participants were selected from each stratum using systematic random sampling techniques.

### Data collection tools and techniques

An interviewer-administered pretested and validated semi-structured questionnaire adapted from available literature (29, 31, 34) and modified to the study variables was used to collect the data. The questionnaire was initially developed in English, translated to the local languages (Afan Oromo and Amharic), and then re-translated into English by language experts to check for consistency. The questionnaire consisted of socioeconomic and demographic characteristics, nutritional status, behavioral characteristics, history of previous detentions, duration of imprisonment, clinical conditions and service-related variables, and anthropometric measurements. The data regarding socio-demographic characteristics and related variables were collected by trained data collectors and supervisors using interviewer-administered pretested questionnaires. Behavioral characteristics such as smoking cigarettes were gathered using “Yes/No” questions. Prisoners were classified as smokers if they smoked one or more cigarettes per day (35). Similarly, Yes/No questions were used to assess the presence of acute or chronic diseases such as tuberculosis (TB).

### Measurement of variables

#### Undernutrition

Undernutrition was considered in the adult prisoners whose body mass index (BMI) was less than 18.5 kg/m^2^ (36, 37).

#### Anthropometric measurements

The weight and height of the participants were measured using a digital standing weight scale and stadiometer (Detecto, United Kingdom) which measures weight and height together. The weight scale was calibrated to zero before measuring each participant and the accuracy of the instrument was checked by measuring the weight of a known object. The accuracy of the stadiometer was also checked by measuring the height of an object with a known height. The weight and height measurements were taken while wearing only light clothing, bare feet, and no headwear. The height was measured while respondents were standing erect against the stadiometer with the shoulder, buttock, calf and heels touching the stadiometer and eyes looking straight ahead (Frankfurt plane) so that the line of sight was perpendicular to the body. Both weight and height were recorded to the nearest 0.1 kg and 0.1 cm, respectively (30, 31, 37). The BMI was calculated by a person’s weight in kilograms divided by the square of height in meters.

#### Dietary diversity score

Individual dietary diversity score (IDDS) was measured after dietary intake data were collected using a 24 h dietary recall method. Any type of food that was consumed by participants within 24 h before the time of data collection was recorded. A set of 10 food groups were used to guide the scoring per the food items consumed. Participants received a “1” point if they consumed a minimum of one food within each sub-group, and a “0” if they did not (38). The scores were summed up to get the total IDDS. Finally, prisoners with a score of 5 and above were categorized as having a diversified diet and those with a score of less than 5 were classified as having a non-diversified diet (39).

#### Depression

Depression was assessed using the Patient Health Questionnaire-nine (PHQ-9) with a five-point severity scale. The tool has strong psychometric properties as evidenced by its validity and reliability, and a score of 10 or more on this scale is reported to have a sensitivity of 88% and a specificity of 88% for major depressive disorder (40). The total score was computed by adding the scores of all nine items on the scale. The depressive symptoms on PHQ-9 were rated on a scale ranging from “0” (not at all) to “3” (nearly every day). The total score ranges from 0–27. The scores represent 0–4 considered as none/minimal, 5–9 as mild, 10–14 as moderate, 15–19 as moderately severe, and 20–27 as severe depression (41). The participants were considered in a state of depression if they scored five and above.

#### Khat chewing

Khat chewing was measured considering both lifetime chewing duration (in years) and time spent in a single chewing session (in hours). Participants who used khat for more than five years and chewed for more than four hours in a single chewing session before they were incarcerated were considered khat chewers (31).

### Data quality management

A pretested and validated semi-structured data collection tool was adapted to ensure data quality. Two days training were given to data collectors and supervisors on the objectives of the study, the contents of data collection tools, the anthropometric measurements, and how to collect and record data appropriately. A pretest was conducted on 5% of the sample size in a similar study population before the actual data collection period to check for the reliability and validity of data collection tools. The questionnaires were reviewed and checked for completeness and consistency, and necessary amendments were made based on the results of the pretest. The collected data were carefully checked for completeness, accuracy and consistency by supervisors and the principal investigator on daily basis. Double data entry was done by two individuals to minimize errors.

### Data processing and analysis

The collected data were cleaned, coded and entered into Epi-Data version 3.1 and analyzed using Statistical Package for Social Sciences (SPSS) version 20.0 software. Texts, tables and figures were used to display descriptive and summary statistics. The binary logistic regression analysis was conducted to identify the determinants of undernutrition. Initially, the bivariate logistic analysis was conducted to determine the candidate variables for the multivariate logistic analysis. All variables with a *p*-value of less than 0.25 in the bivariate logistic analysis were fitted into the multivariate logistic analysis to identify factors significantly associated with undernutrition. The variance inflation factor (VIF) was used to check the existence of multicollinearity among variables. All variables were observed with VIF <2, showing the non-existence of multicollinearity. The logistic regression goodness of fit of the model was checked using the Hosmer and Lemeshow statistical test and indicated a good fit for the model at a *p*-value of 0.793. Both crude and adjusted odds ratios with a 95% CI were calculated to show the strength of the association, and a *p*-value <0.05 was used to declare statistical significance.

## Results

### Socio-demographic characteristics of study participants

A total of 479 adult prisoners were enrolled in the study, giving a response rate of 96.4%. The mean (SD) age of the study participants was 39.64 (±12.21) years. Among the prisoners, more than one-fourth (27.76%) were in the age group of 30–39 years. The majority (62.2%) of the prisoners were males. In this study, nearly two-thirds (64.3%) of the prisoners were ever married and 62.84% had never received any level of formal education. More than half (54.5%) of the prisoners were rural residents and 48.43% were farmers before imprisonment (Table 1).

**Table 1:** Socio-demographic characteristics of the study participants in Fiche town prison, central Ethiopia, 2020 (*n* = 479).

Variables	Category	Frequency (*N*)	Percentage (%)
Sex	Male	298	62.20
	Female	181	37.80
Age (in years)	18–29	119	24.84
	30–39	133	27.76
	40–49	113	23.60
	>49	114	23.80
Religion	Orthodox	322	67.22
	Protestant	90	18.79
	Muslim	55	11.48
	Others	12	2.51
Marital status	Never married	308	35.70
	Ever married	171	64.30
Educational status	No formal education	301	62.84
	Primary education	76	15.87
	Secondary education	67	13.99
	Tertiary education	35	7.30
Previous occupation	Farmer	232	48.43
	Merchant	113	23.59
	Government employee	53	11.06
	Daily laborer	33	6.89
	Jobless	48	10.03
Previous residence	Rural	261	54.50
	Urban	218	45.50

### Detention conditions of prisoners

Among the prisoners, 46.35% were held in prison for 25 months and above. The majority (86.85%) had no history of previous detentions. Almost all (98.75%) prisoners were sleeping in a group. The majority (78.5%) of the prisoners were involved in income-generating activities in the prison. More than two-thirds (72.23%) of the prisoners were not visited by their families and more than three-quarters (80.17%) did not receive any financial support from their families or significant others (Table 2).

**Table 2:** Detention conditions of the prisoners in Fiche town prison, central Ethiopia, 2020 (*n* = 479).

Variables	Category	Frequency (*N*)	Percentage (%)
Duration of detention (in months)	6–12	115	24.01
	13–24	142	29.64
	25–59	114	23.80
	>59	108	22.55
History of previous detention	Yes	63	13.15
	No	416	86.85
Sleeping condition	Individual	6	1.25
	In a group	473	98.75
Engaged in income-generating work in the prison	No	103	21.50
	Yes	376	78.50
Family visits	No	346	72.23
	Yes	133	27.77
Financial support	No	384	80.17
	Yes	95	19.83

### Behavioral, nutritional and health-related characteristics

The majority (86.22%) of the prisoners were nonsmokers. Less than one-third (29.85%) of the prisoners had a good dietary diversity score (DDS), and three-fourths of prisoners ate three times a day. More than three-fourths (77.45%) of the prisoners had food sources other than the prison. Regarding health-related characteristics, 13.99 and 3.76% of the prisoners had a self-reported current illness and a history of TB treatment in the last 12 months, respectively. In the current study, 5.43% and 1.46% of the prisoners had moderately severe and severe depression during the survey, respectively and were linked to nearby health facilities for further evaluation and management (Table 3).

**Table 3:** Behavioral, nutritional and health-related characteristics of the study participants in Fiche town prison, central Ethiopia, 2020 (*n* = 479).

Variables	Category	Frequency (*N*)	Percentage (%)
Smoking	No	413	86.22
	Yes	66	13.78
Alcohol use	No	396	82.67
	Yes	83	17.33
Chew khat	No	463	96.66
	Yes	16	3.34
Meal frequency (per day)	Once	6	1.25
	Twice	56	11.70
	Three times	345	72.03
	Four or more times	72	15.02
Food sources other than the prison	No	108	22.55
	Yes	371	77.45
Dietary diversity (DD)	Low	43	8.98
	Medium	293	61.17
	Good	144	29.85
Self-reported current illness	No	412	86.01
	Yes	67	13.99
History of TB treatment in the last 12 months	No	461	96.24
	Yes	18	3.76
History of medical treatment for the last 2 weeks	No	460	96.03
	Yes	19	3.97
Depression	None/minimal	317	66.18
	Mild	92	19.21
	Moderate	37	7.72
	Moderately severe	26	5.43
	Severe	7	1.46

TB, Tuberculosis.

### Magnitude of undernutrition

**Figure 1:**
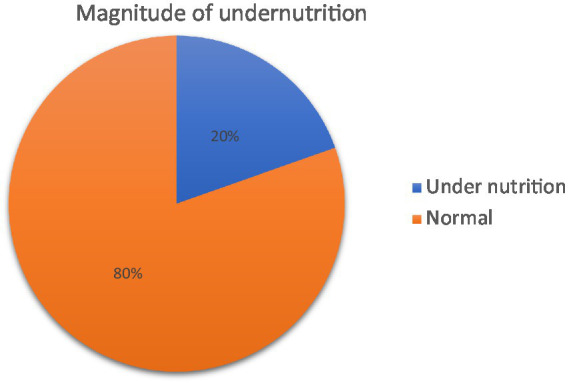
Magnitude of undernutrition among adult prisoners in Fiche town prison, central Ethiopia, 2020 (*n* = 479).

**Figure 2:**
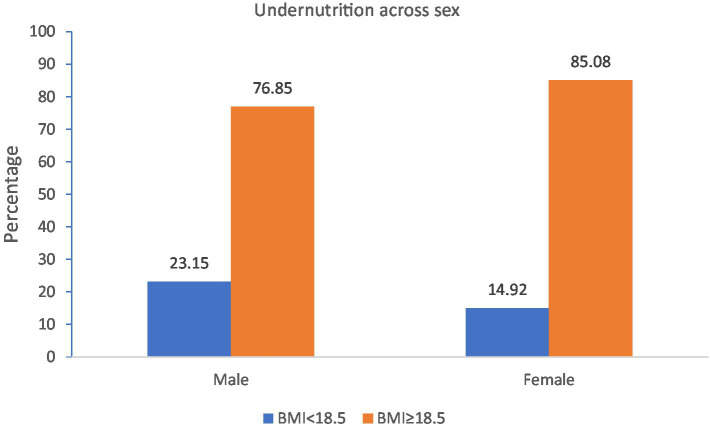
Magnitude of undernutrition across sex among adult prisoners in Fiche town prison, central Ethiopia, 2020 (*n* = 479).

The overall magnitude of undernutrition among adult prisoners in this study was found to be 20% (95% CI: 16.5–23.6) (Figure 1). Among undernourished prisoners, 6.7% were moderately undernourished. About 23.15% of the male prisoners were undernourished in the study (Figure 2).

### Factors associated with undernutrition

In the bivariable binary logistic analysis, sex, financial support, duration of imprisonment, income-generating work in the prison, dietary diversity, and depression were significantly associated with undernutrition. The odds of undernutrition among female prisoners were reduced by 49% (AOR = 0.51; 95% CI: 0.26, 0.98). The odds of undernutrition among prisoners with financial support were reduced by 64% (AOR = 0.36; 95% CI: 0.15, 0.87). Prisoners who were incarcerated for 25 to 59 months (AOR = 3.07; 95% CI: 1.33, 7.04) and for greater than 59 months (AOR = 4.56; 95% CI: 2.00, 10.45) were more likely to be undernourished compared to prisoners who were detained for 6 to 12 months. The odds of undernutrition among prisoners engaged in income-generating work were reduced by 73% (AOR = 0.27; 95% CI: 0.15, 0.47). Similarly, the risk of developing undernutrition among prisoners with medium dietary diversity (AOR = 0.35; 95% CI: 0.15, 0.80) and good dietary diversity (AOR = 0.23; 95% CI: 0.08, 0.61) was reduced by 65 and 77%, respectively. Moreover, the odds of undernutrition among prisoners with mild and moderate depression and moderately severe and severe depression were (AOR = 1.9; 95% CI: 1.05, 3.45) and (AOR = 2.78; 95% CI: 1.17, 6.60), respectively (Table 4).

**Table 4:** Factors associated with undernutrition among adult prisoners in Fiche town prison, central Ethiopia, 2020 (*n* = 479).

Variables	Under nutrition		COR (95% CI)	*p*-value	AOR (95% CI)	*p*-value
	Yes (*n* = 96) *N* (%)	No (*n* = 383) *N* (%)				
**Sex**						
Male	69 (23.15)	229 (76.85)	1		1	
Female	27 (14.92)	154 (85.08)	1.01 (0.64–1.61)	0.07	0.51(0.26–0.98)^a^	**0.04**
**Age category (in years)**						
18–29	24 (20.17)	95 (79.83)	1		1	
30–39	21 (15.79)	112 (84.21)	0.78 (0.39–1.41)	0.19	0.63 (0.28–1.40)	0.26
40–49	20 (17.70)	93 (82.30)	0.85 (0.44–1.64)	0.24	0.84 (0.31–2.03)	0.70
>49	31 (27.19)	83 (72.81)	1.47 (0.80–2.72)	0.10	0.98 (0.39–2.42)	0.97
**Marital status**						
Never married	65 (21.10)	243 (78.90)	1		1	
Ever married	31 (18.13)	140 (81.87)	0.82 (0.51–1.33)	0.21	1.06 (0.60–1.88)	1.06
**Financial support**						
No	86 (22.39)	298 (77.61)	1		1	
Yes	10 (10.53)	85 (89.47)	0.41 (0.20–0.82)	0.23	0.36 (0.15–0.87)^a^	**0.02**
**Food sources other than the prison**						
No	24 (22.22)	84 (77.78)	1		1	
Yes	72 (19.41)	299 (80.59)	0.84 (0.50–1.42)	0.63	0.82 (0.39–1.68)	0.58
**Duration of imprisonment (in months)**						
6–12	12 (10.43)	103 (89.57)	1		1	
13–24	17 (11.97)	125 (88.03)	1.16 (0.53–2.55)	0.68	1.28 (0.55–3.02)	0.56
25–59	(27 23.68)	87 (76.32)	2.66 (1.27–5.57)	0.20	3.07 (1.33–7.04)^a^	**0.00**
>59	40 (37.04)	68 (62.96)	5.05 (2.47–10.31)	0.00	4.56 (2.00–10.45)^a^	**0.00**
**Family visits**						
No	69 (19.94)	277 (80.06)	1		1	
Yes	27 (20.30)	106 (79.70)	1.02 (0.62–1.68)	0.29	0.66 (0.41–1.20)	0.36
**Income-generating work in the prison**						
No	41 (39.81)	62 (60.19)	1		1	
Yes	55 (14.63)	321 (85.37)	0.26 (0.16–0.42)	0.02	0.27 (0.15–0.47)^a^	**0.00**
**Dietary diversity (DD)**						
Low	23 (53.49)	20 (46.51)	1		1	
Medium	241 (82.25)	52 (17.75)	0.24 (0.12–0.47)	0.14	0.35 (0.15–0.80)^a^	**0.01**
Good	120 (83.33)	24 (16.67)	0.22 (0.10–0.46)	0.21	0.23 (0.08–0.61)^a^	**0.00**
**Self-reported current illness**						
No	57 (13.83)	355 (86.17)	1	0.24	1	
Yes	28 (41.79)	39 (58.21)	4.47(2.55–7.83)		5.20 (2.69–10.07)	0.07
**Depression**						
No/minimal	49 (15.46)	268 (84.54)	1		1	
Mild & moderate	37 (28.68)	92 (71.32)	2.05 (1.24–3.39)	0.03	1.90 (1.05–3.45)^a^	**0.01**
Moderately severe & severe	10 (30.30)	23 (69.70)	2.83 (1.39–5.79)	0.02	2.78 (1.17–6.60)^a^	**0.00**

a Significantly associated variables at a *p*-value <0.05. The bold values are to indicate/show significantly associated variables that had *p*-value <0.05.

## Discussion

This study was conducted to determine the magnitude of undernutrition and associated factors among adult prisoners in Fiche town prison, central Ethiopia. Sex, financial support, duration of imprisonment, income-generating work in the prison, dietary diversity, and depression were predictors of undernutrition among adult prisoners in Fiche town prison.

In the current study, the overall magnitude of undernutrition among adult prisoners was found to be 20%. This finding is consistent with the studies conducted in Ethiopian prisons: Mizan (18.6%) (31) and Butajira (23.2%) (30). However, this finding is lower than the findings of the studies conducted in Tigray prison, Ethiopia (25.2%) (29), Kality prison, Addis Ababa, Ethiopia (43%) (42) and Antanimora prison, Madagascar (38.4%) (28). This disparity might be because of the difference in characteristics of the study participants. The current study was conducted among all prisoners, but the study conducted in Kality prison was among prisoners living with HIV/AIDS; increasing body demand for nutrients, diminishing body stores, reducing food intake, and adversely affecting nutrient absorption and metabolism (27), whereas a study from Antanimora prison was among female prisoners. In disparity, this finding is higher than the studies from New Guinea (5%) (23) and Nigeria (4%) (43). This discrepancy might be, due to the difference in the study setting, the number of prisoners in the study area and the socioeconomic characteristics of study participants.

In this study, female prisoners were less likely to be undernourished compared to male prisoners. This might be due to the coordinated effort to improve the situation by the female prisoner’s administrative office that put female prisoners in a relatively better state of health and nutrition than male prisoners in the study. In addition, women can consume more energy than they expend and accumulate fat more effectively, making them more efficient at energy conservation (44). An additional explanation could be that the federal government has coordinated efforts to ameliorate the situation of female inmates, resulting in better health and nutrition than male inmates (42, 45).

Prisoners who had financial support were less likely to be at risk of developing undernutrition compared to their counterparts. This finding is supported by the studies conducted in Tigray, Butajira and Mizan prisons in Ethiopia which showed prisoners with financial support were less likely to be undernourished (29–31). This could be because prisoners who have financial support can afford to buy meals from outside the prison and thus have better access to adequate and diverse food. Similarly, prisoners who were engaged in income-generating work in the prison were less likely to be undernourished compared to prisoners who were not. This could be because the foods in many prisons are insufficient to provide the body with the essential energy (28), and prisoners who are engaged in income-generating work can afford to buy meals from outside the prison for their caloric needs.

In the present study, the duration of imprisonment was significantly associated with undernutrition. Prisoners who had been imprisoned for a longer time were more likely to be undernourished compared to prisoners who had been imprisoned for a shorter time. This finding is in line with the studies conducted in Tigray, Butajira and Mizan prisons in Ethiopia (29–31). Moreover, this finding is also supported by the study conducted among female prisoners in Antanimora prison, Madagascar (28). The evidence from the study has shown that prison foods are frequently nutritionally deficient, and prisoners who have been detained for a long time are exposed to these nutritionally deficient foods regularly, potentially leading to undernutrition (33).

The finding of this study revealed that prisoners with medium and good dietary diversity were less likely to be undernourished compared to prisoners with lower dietary diversity. Previous literature showed a strong association between dietary diversity and nutritional status (46–48). This is because dietary diversity is associated with food availability and nutrient intake and is an important factor in nutritional outcomes (49).

The finding of the current study further revealed that depression was significantly associated with undernutrition. The prisoners who had depression were more likely to be undernourished compared to their counterparts. The finding of this study is in line with the study conducted in Mizan Prison Institute, Ethiopia (31). This could be, due to depression, which can affect people’s appetite and lead to a reduction in food intake, resulting in significant weight loss and undernutrition (41, 50). The evidence from the studies also showed that malnutrition increases the risk of depression (51).

### Strengths and limitations

The primary data were gathered from the study participants and used in this study to determine the magnitude of undernutrition. The calibrated and standardized anthropometric instruments were used to minimize measurement error. The study provides research-based relevant data on the magnitude of undernutrition and identifies its determinant factors which could help health policymakers in designing evidence-based preventive measures. Despite these important merits, the study had the following limitations. Primarily, due to the nature of the cross-sectional study design, it was difficult to establish the cause-effect relationship between the variables. Secondly, the BMI used to measure undernutrition was one of the limitations since it is not sensitive enough to recognize small clinically significant weight loss. The self-reported questionnaires were prone to social desirability bias. Moreover, recall bias was another limitation since some questions were asked about the events that occurred 24 h back. This was minimized by probing the respondents about the events.

## Conclusion

The magnitude of undernutrition among adult prisoners was found to be high. One in five prisoners was undernourished in Fiche town prison, alarming the need for urgent and appropriate interventions. Sex, financial support, duration of imprisonment, income-generating work in the prison, dietary diversity, and depression were predictors of undernutrition. Therefore, the prison authorities should ensure that prisoners have access to healthy food options and diversified diets, as well as implementing early screening and treatment of depression are crucial in reducing the burden of undernutrition and related health conditions. In addition, encouraging prisoners to participate in income-generating activities within the prison is recommended in the study setting. Moreover, prisoners who lack social support and have been incarcerated for a prolonged period of time should be given special attention.

## Data availability statement

The original contributions presented in the study are included in the article/supplementary material, further inquiries can be directed to the corresponding author.

## Ethics statement

The studies involving human participants were reviewed and approved by Haramaya University, College of Health and Medical Sciences’ Institutional Health Research and Ethics Review Committee (IHRERC). The patients/participants provided their written informed consent to participate in this study.

## Author contributions

MW, AS, and BA conceived, designed, acquired data, analyzed, and interpreted the findings. IK, AG, SH, AM, and BA revised and provided critical intellectual feedback. IK and SH drafted the manuscript. All authors contributed to the article and approved the submitted version.

## Funding

This study was financially supported by Haramaya University. The authors report that the funding body had no role in the study selection, data collection, analysis, conclusion, and interpretation.

## Conflict of interest

The authors declare that the research was conducted in the absence of any commercial or financial relationships that could be construed as a potential conflict of interest.

## Publisher’s note

All claims expressed in this article are solely those of the authors and do not necessarily represent those of their affiliated organizations, or those of the publisher, the editors and the reviewers. Any product that may be evaluated in this article, or claim that may be made by its manufacturer, is not guaranteed or endorsed by the publisher.

## Abbreviations

AOR: Adjusted Odds Ratio
CI: Confidence Interval
COR: Crude Odds Ratio
DDS: Dietary Diversity Score
TB: Tuberculosis
WHO: World Health Organization

## References

[1] Awosan K Ibrahim M Essien E Yusuf A Okolo A. Dietary pattern, lifestyle, nutrition status and prevalence of hypertension among traders in Sokoto central market, Sokoto, Nigeria. Int J Nutr Metab. (2014) 6:9–17. doi: 10.5897/ijnam2013.0158

[2] Ferede YM Derso T Sisay M. Prevalence of malnutrition and associated factors among older adults from urban and rural residences of Metu district. BMC Nutr. (2022) 8:52. doi: 10.1186/s40795-022-00532-9, PMID: 35637535 PMC9150330

[3] Tessfamichael D Gete AA Wassie MM. High prevalence of undernutrition among elderly people in northwest Ethiopia: a cross-sectional study. J Nutr Health Food Sci. (2014) 2:1–5. doi: 10.15226/jnhfs.2014.00131

[4] Smith ML Bergeron CD Lachenmayr S Eagle LA Simon JR. A brief intervention for malnutrition among older adults: stepping up your nutrition. Int J Environ Res Public Health. (2020) 17:3590. doi: 10.3390/ijerph17103590, PMID: 32443789 PMC7277589

[5] Raynaud-Simon A Revel-Delhom C Hébuterne X. Clinical practice guidelines from the French health high authority: nutritional support strategy in protein-energy malnutrition in the elderly. Clin Nutr. (2011) 30:312–9. doi: 10.1016/j.clnu.2010.12.003, PMID: 21251732

[6] Saunders J Smith T. Malnutrition: causes and consequences. Clin Med. (2010) 10:624–7. doi: 10.7861/clinmedicine.10-6-624, PMID: 21413492 PMC4951875

[7] Leach B Goodwin S. Preventing malnutrition in prison. Nurs Stand. (2014) 28:50–6. doi: 10.7748/ns2014.01.28.20.50.e7900 24422845

[8] Kavithe KR Dorcus MD Maoga WN. Dietary intake and factors affecting food service of male prisoners living with human immunodeficiency virus at selected prisons in Kenya. Int J Nutr Metab. (2018) 10:6–15. doi: 10.5897/ijnam2017.0229

[9] Martins VJ Toledo Florêncio TM Grillo LP do Carmo Franco M Martins PA Clemente AP. Long-lasting effects of undernutrition. Int J Environ Res Public Health. (2011) 8:1817–46. doi: 10.3390/ijerph8061817, PMID: 21776204 PMC3137999

[10] Elia M. The ‘MUST’report. Nutritional screening of adults: a multidisciplinary responsibility. (2003). Available at: http://eprints.soton.ac.uk/id/eprint/362499

[11] Naharci MI Katipoglu B Tasci I. Association of anticholinergic burden with undernutrition in older adults: a cross-sectional study. Nutr Clin Pract. (2022) 37:1215–24. doi: 10.1002/ncp.10821, PMID: 34994474

[12] Jacobson J Heard C Fair H. Prison: evidence of its use and over-use from around the world. University of London, England. (2017).

[13] TIO Justice. Global prison trends 2019: penal reform international 2019; (2019). Available at: https://knowledge.tijthailand.org/en/publication/detail/globalprisontrends#book/ (Accessed May 11, 2022)

[14] Nations U. Ethiopia prison factsheet: the United Nations office on drugs and crime; (2021). Available at: https://www.unodc.org/documents/easternafrica/ETHIOPIA/UNODC_Ethiopia_Prison_Factsheet_FINAL_14_June_2021.pdf (Accessed May 11, 2022)

[15] Sarkin J. Prisons in Africa: an evaluation from a human rights perspective. Sur Int Hum Rights J. (2009) 9:22–49. doi: 10.1590/s1806-64452008000200003, PMID: 27383337

[16] Minayo MCS Ribeiro AP. Health conditions of prisoners in the state of Rio de Janeiro, Brazil. Cien Saude Colet. (2016) 21:2031–40. doi: 10.1590/1413-81232015217.08552016, PMID: 27383337

[17] Graham L Fischbacher CM Stockton D Fraser A Fleming M Greig K. Understanding extreme mortality among prisoners: a national cohort study in Scotland using data linkage. Eur J Public Health. (2015) 25:879–85. doi: 10.1093/eurpub/cku252, PMID: 25678604

[18] Assembly UNG. Universal declaration of human rights, vol. 3381 Department of State, United States of America. United Nations, USA. (1949).

[19] Assembly UNG. United Nations standard minimum rules for the treatment of prisoners (the Nelson Mandela rules). The resolution was adopted by the General Assembly on 17 December 2015. UN Doc. UN Doc A/RES/70/175. (2016) 8.

[20] Gatherer A Møller L. Health in prisons: a WHO guides to the essentials in prison health. WHO regional office Europe; (2007).

[21] Herbert K Plugge E Foster C Doll H. Prevalence of risk factors for non-communicable diseases in prison populations worldwide: a systematic review. Lancet. (2012) 379:1975–82. doi: 10.1016/S0140-6736(12)60319-5, PMID: 22521520

[22] Winetsky DE Almukhamedov O Pulatov D Vezhnina N Dooronbekova A Zhussupov B. Prevalence, risk factors and social context of active pulmonary tuberculosis among prison inmates in Tajikistan. PLoS One. (2014) 9:e86046. doi: 10.1371/journal.pone.008604623, PMID: 24465861 PMC3896449

[23] Gould C Tousignant B Brian G McKay R Gibson R Bailey K. Cross-sectional dietary deficiencies among a prison population in Papua New Guinea. BMC Int Health Hum Rights. (2013) 13:21. doi: 10.1186/1472-698X-13-21, PMID: 23601963 PMC3637570

[24] Aké-Tano O Konan EY Tetchi EO Ekou FK Ekra D Coulibaly A. Beriberi, recurrent nutritional disease in a detention house in Côte-d'Ivoire. Bull Soc Pathol Exot. (2011) 104:347–51. doi: 10.1007/s13149-011-0136-6, PMID: 21336653

[25] Ashdown J James M. Women in detention. Int Rev Red Cross. (2010) 92:123–41. doi: 10.1017/S1816383110000226

[26] Khoda Bakhshi Fard A Safarian M Rostami S Zamani S Mazidi M Arabi M. Evaluation of the nutritional status using the anthropometric indices and dietary intakes in the central prison of Mashhad. J Biol Today’s World. (2014) 3:266–70. doi: 10.15412/J.JBTW.01031203

[27] Daniel M Mazengia F Birhanu D. Nutritional status and associated factors among adult HIV/AIDS clients in Felege Hiwot referral hospital, Bahir Dar, Ethiopia. Sci J Pub Health. (2013) 1:24–31. doi: 10.11648/j.sjph.20130101.14

[28] Ravaoarisoa L Pharlin AH Andriamifidison NZR Andrianasolo R Rakotomanga JDM Rakotonirina J. Nutritional status of female prisoners in Antanimora prison, Madagascar. Pan Afr Med J. (2019) 33:119. doi: 10.11604/pamj.2019.33.119.18170, PMID: 31489097 PMC6711675

[29] Abera SF Adane K. One-fourth of the prisoners are underweight in northern Ethiopia: a cross-sectional study. BMC Public Health. (2017) 17:449. doi: 10.1186/s12889-017-4410-9, PMID: 28506311 PMC5433041

[30] Melis T. Prevalence of Undernutrition and its associated factors among adult prison inmates in Butajira prison, southern Ethiopia, 2020. J Nutr Disord. (2021) 11:650.

[31] Wondimu W Girma B Sinaga M Taye A. Undernutrition and associated factors among incarcerated people in Mizan prison institute, Southwest Ethiopia. PLoS One. (2021) 16:e0251364. doi: 10.1371/journal.pone.0251364, PMID: 33974638 PMC8112703

[32] WHO (World Health Organization). Prisons and health Regional Office Europe, World Health Organization. Denmark: WHO Regional Office. (2014).

[33] Sawyer W. Food for thought: prison food is a public health problem. Prison policy initiative. The Ladies of Hope Ministries, USA. (2017).

[34] De Coninck G Okenge Ngongo L Ilunga Ilunga F Albert A Giet D Kalonji MP. Nutritional status of inmates in the central prison of Mbuji-Mayi, Democratic Republic of Congo. Int J Nutr. (2020) 6:11–20. doi: 10.14302/issn.2379-7835.ijn-21-3926

[35] Hartwig C Stöver H Weilandt C. Report on tobacco smoking in prison. Directorate-General for Health and Consumers. Drug policy and harm Reduction. University of Bremen, German. (2008) 1–36.

[36] World Health Organization. Physical status: the use and interpretation of anthropometry: report of a WHO expert committee. Geneva; 1995. World Health Organ Tech Rep Ser. (1995) 854:312–44. 8594834

[37] Nubé M Van Den Boom GJM. Gender and adult undernutrition in developing countries. Ann Hum Biol. (2003) 30:520–37. doi: 10.1080/0301446031000119601 12959894

[38] FAO and FHI 360. Minimum dietary diversity for women. A guide to measurement In: Minimum dietary diversity for women: A guide for measurement. Rome, Italy: FAO (2016). 82.

[39] FAO Dietary Assessment. A resource guide to method selection and application in low resource settings. Rome, Italy: FAO (2018). 152 p.

[40] Kroenke K Spitzer RL Williams JB. The PHQ-9: validity of a brief depression severity measure. J Gen Intern Med. (2001) 16:606–13. doi: 10.1046/j.1525-1497.2001.016009606.x, PMID: 11556941 PMC1495268

[41] Abdu Z Kabeta T Dube L Tessema W Abera M. Prevalence and associated factors of depression among prisoners in Jimma town prison, south west Ethiopia. Psychiatry J. (2018) 2018:5762608. doi: 10.1155/2018/5762608, PMID: 30018974 PMC6029452

[42] Kassa T Alle A Tesfu M. Assessment of nutritional status and associated factors among prisoners living with HIV/AIDS in Kality prison, Addis Ababa, Ethiopia. J AIDS Clin Res. (2017) 8:1–6. doi: 10.4172/2155-6113.1000703

[43] Akinlotan J Nupo S Olorode O. Assessment of nutritional status of inmates in Oyo state, Nigeria. J Multidiscip Res. (2010) 2:68–75.

[44] Wu BN O'Sullivan AJ. Sex differences in energy metabolism need to be considered with lifestyle modifications in humans. J Nutr Metab. (2011) 2011:391809:1–6. doi: 10.1155/2011/391809, PMID: 21773020 PMC3136178

[45] Weldeyohannes BT. Reforming prison reforming prison policy to improve women-specific health and Omen-specific health and sanitary care conditions of prisons in Ethiopia. Wm Mary J Women L. (2017) 24:101. doi: 10.2139/ssrn.2661169

[46] Maila G Audain K Marinda PA. Association between dietary diversity, health and nutritional status of older persons in rural Zambia. South Afr J Clin Nutr. (2019) 34:34–9. doi: 10.1080/16070658.2019.1641271

[47] Olatunji E Obonyo C Wadende P Were V Musuva R Lwanga C. Cross-sectional Association of Food Source with food insecurity, dietary diversity and body mass index in Western Kenya. Nutrients. (2021) 14:121. doi: 10.3390/nu14010121, PMID: 35010996 PMC8747304

[48] Lee SJ Ryu HK. Relationship between dietary intakes and the double burden of malnutrition in adults of Malang, Indonesia: an exploratory study. Nutr Res Pract. (2018) 12:426–35. doi: 10.4162/nrp.2018.12.5.426, PMID: 30323910 PMC6172173

[49] Nithya D Bhavani R. Dietary diversity and its relationship with nutritional status among adolescents and adults in rural India. J Biosoc Sci. (2018) 50:397–413. doi: 10.1017/S0021932017000463, PMID: 28967344

[50] Vafaei Z Mokhtari H Sadooghi Z Meamar R Chitsaz A Moeini M. Malnutrition is associated with depression in rural elderly population. J Res Med Sci. (2013) 18:S15–9. doi: 10.1186/s12877-021-02535-w, PMID: 23961277 PMC3743311

[51] Alam MR Karmokar S Reza S Kabir MR Ghosh S Mamun MAA. Geriatric malnutrition and depression: evidence from elderly home care population in Bangladesh. Prev Med Rep. (2021) 23:101478. doi: 10.1016/j.pmedr.2021.101478, PMID: 34458076 PMC8377374

